# Use of Selective Cyclooxygenase-2 Inhibitors, Other Analgesics, and Risk of Glioma

**DOI:** 10.1371/journal.pone.0149293

**Published:** 2016-02-12

**Authors:** Corinna Seliger, Christoph R. Meier, Claudia Becker, Susan S. Jick, Ulrich Bogdahn, Peter Hau, Michael F. Leitzmann

**Affiliations:** 1 Department of Neurology and Wilhelm Sander-NeuroOncology Unit, Regensburg University Hospital, Regensburg, Germany; 2 Basel Pharmacoepidemiology Unit, Division of CIinical Pharmacy and Epidemiology, Department of Pharmaceutical Sciences, University of Basel, Basel, Switzerland; 3 Boston Collaborative Drug Surveillance Program, Boston University School of Public Health, Boston, Massachusetts, United States of America; 4 Hospital Pharmacy, University Hospital Basel, Basel, Switzerland; 5 Department of Epidemiology and Preventive Medicine, University of Regensburg, Regensburg, Germany; University of Calgary, CANADA

## Abstract

**Background:**

Selective cyclooxygenase-2 (COX-2) inhibitors are analgesic, antipyretic, and anti-inflammatory drugs. They have been found to inhibit the development of glioma in laboratory investigations. Whether these drugs reduce the risk of glioma incidence in humans is unknown.

**Methods:**

We conducted a matched case-control analysis using the U.K.-based Clinical Practice Research Datalink (CPRD). We identified 2,469 cases matched to 24,690 controls on age, sex, calendar time, general practice, and number of years of active history in the CPRD prior to the index date. We conducted conditional logistic regression analyses to determine relative risks, estimated as odds ratios (ORs) with 95% confidence intervals (CIs) of glioma in relation to use of selective COX-2 inhibitors, adjusted for several confounding variables.

**Results:**

Use of selective COX-2 inhibitors was unrelated to risk of glioma (adjusted OR for 1–9 versus 0 prescriptions = 1.02; 95% CI = 0.92–1.13, 10–29 versus 0 prescriptions = 1.01; 95% CI = 0.80–1.28, ≥30 versus 0 prescriptions = 1.16; 95% CI = 0.86–1.55). Trends for increasing numbers of prescriptions for other non-steroidal anti-inflammatory drugs (NSAIDs), and non-NSAID analgesics were also not associated with glioma risk.

**Conclusion:**

Further epidemiologic studies are needed to confirm the null relation of use of selective COX-2 inhibitors to glioma risk and to explain the discrepancy between laboratory investigations and our observational study. Impact: Use of selective COX-2 inhibitors is unrelated to glioma risk.

## Introduction

Malignant gliomas are highly aggressive tumours of the central nervous system [1]. Glioblastoma is the most common and malignant type of glioma and it is associated with a median overall survival of 15 months despite aggressive therapy [1]. In contrast to many other cancers, there are only a few established risk factors for gliomas including increasing age, male gender, Caucasian ethnicity, rare genetic syndromes, and a high-level of ionizing radiation [2].

Selective COX-2 inhibitors are commonly used analgesics frequently prescribed for rheumatoid arthritis, dysmenorrhoea, or acute pain. Besides their analgesic, anti-inflammatory, and antipyretic effects, they have been found to inhibit or kill glioma cells [3–9], increase radio-sensitivity [10–15], reduce angiogenesis [14, 16–19], and stimulate anti-tumour immune reactions *in vitro* and in established animal models [16, 20–23]. Clinical trials have tested selective COX-2 inhibitors as adjuvant therapy for glioma, but results have been inconclusive thus far [17, 24–32].

Although selective COX-2 inhibitors reduce gliomagenesis in vitro and in vivo [33], the association between these agents and the risk of glioma has not yet been investigated in observational studies. Prior studies on NSAIDS and glioma, including one study using CPRD data [34], reported null [34–38], inverse [39–42], or positive [43, 44] associations, but those studies did not examine the effect of selective COX-2 inhibitors specifically. The lack of studies on the effect of selective COX-2 inhibitors as a distinct group and the promising biological evidence therefore prompted us to analyse the relation of selective COX-2 inhibitors use to the risk of glioma.

## Patients and Methods

### Data source

The Clinical Practice Research Datalink (CPRD) is a primary care database that holds patient information from around 600 general practices representative of the U.K. population with respect to age, sex, and geographic distribution. Standard coding systems are used by general practitioners to record data on patient diagnoses, prescriptions, hospital admissions, and referrals to specialists. Data from office computers are transferred to electronic patient records, as has been described previously [45]. The reliability of diagnostic coding in the CPRD database has been thoroughly validated [46, 47].

The current study was approved by the Independent Scientific Advisory Committee of the CPRD (protocol-number: 15_170). Patient records/information was anonymized and de-identified prior to analysis.

### Study population

#### Case definition

We identified patients with newly diagnosed glioma between 1995 and 2015 using READ codes that are based on the WHO classification of glioma [48] as previously described [49]. To validate glioma diagnoses, we used supporting codes such as codes for procedures, radio- or chemotherapy, and referrals to oncology clinics. Only patients younger than 90 years of age were included in the study population. The ‘index date’ was defined as the date of the first glioma diagnosis minus one year. We shifted the date of diagnosis back by one year for cases and controls to account for the lag time between disease development and detection/diagnosis, to account for possible changes in medication use prior to the detection and diagnosis date of glioma, to control for changes in analgesic treatment, and to account for potential earlier detection of pre-existing concomitant diseases in case patients caused by early symptoms of undiagnosed glioma. In order to increase the likelihood of capturing incident cases only and to ensure a sufficient history of medication exposure, we required all patients to have an active history in the database for at least three years prior to the index date. We excluded patients with a history of other concurrent cancers except non-melanoma skin cancer as well as patients with recorded alcoholism or human immunodeficiency virus infection prior to the index date.

#### Control definition

For each case, we identified at random up to 10 controls without a history of glioma from the CPRD base population matched on calendar time (same index date), age (same year of birth +/- 3 years), sex, general practice, and number of years of active history in the database prior to the index date. We assigned for each control the index date of the corresponding matching case. To minimize the risk of using control patients with a possible unrecorded glioma diagnosis, we excluded control patients with a prior history of craniotomy within the last year before the index date. Using a one-year time window was considered sufficient due to the highly invasive phenotype of malignant glioma. We applied the same exclusion criteria to controls as to cases.

### Exposures

We assessed the use of selective COX-2 inhibitors (including etodolac (200–1000 mg/day), meloxicam (7.5–15 mg/day), celecoxib (200–400 mg/day), rofecoxib (12.5–25 mg/day), etoricoxib (30–120 mg/day), valdecoxib (10–40 mg/day), lumiracoxib (100–400 mg/day) and diclofenac (50–150 mg/day)) from the computerized records. COX-2 selectivity can be determined by dividing 50% inhibitory concentrations (IC_50_) of COX-2 by IC_50_ concentrations of COX-1 [50, 51]. Valdecoxib possesses the highest COX-2 selectivity (0.009 [50]), followed by etodolac (0.021[50]), rofecoxib (0.029 [50]), meloxicam (0.032 [50]), celecoxib (0.036 [50]), etoricoxib (0.032 [50]), diclofenac (0.1 [50]), ibuprofen (2.89 [50]), naproxen (>10.87 [50]), and aspirin (>100 [51]). We categorized exposure to selective COX-2 inhibitors based on the number of prescriptions prior to the index date. We categorized subjects who did not receive any selective COX-2 drug prior to the index date as ‘no prior use’ (0 prescriptions, reference). Selective COX-2 users were classified into categories of 1–9, 10–29, or ≥ 30 prescriptions prior to the index date. The number of prescriptions serves as an approximation of exposure duration, since an average prescription covers 45 to 90 days of treatment, depending on the number of tablets (1 or 2) taken per day. In a sensitivity analysis, we considered patients with < 5 prescriptions of selective COX-2 inhibitors as non-users. We also investigated individual use of etodolac, meloxicam, celecoxib, rofecoxib, etoricoxib, valdecoxib, lumiracoxib and diclofenac employing the same exposure categories as for the combined selective COX-2 inhibitor variable where possible.

### Statistical analysis

We conducted conditional logistic regression analyses using the SAS statistical software version 9.4 (SAS Institute Inc, Cary, NC) to determine relative risks, estimated as odds ratios (ORs) with 95% confidence intervals (CIs), of glioma in relation to use of selective COX-2 inhibitors. A two-sided p-value of <0.05 was considered statistically significant. We performed tests of linear trend by modelling the median value of each category as a continuous variable in the multivariate model, the coefficient for which was evaluated using a Wald test.

In univariate analyses, we investigated the influence of various potential confounding variables, including body mass index (BMI, <18.5 kg/m², 18.5–24.9 kg/m², 25.0–29.9 kg/m², ≥30.0 kg/m², unknown), smoking status (never, current, past, unknown) and presence versus absence of specific medical conditions or diseases (congestive heart failure, diabetes, rheumatoid arthritis, allergies (asthma and hay fever), and use of estrogens (0, 1–14, ≥15 prescriptions; women only). We only included variables in the final multivariate analysis that altered the risk estimate of glioma by >10%. We adjusted our final analysis for BMI, smoking, diabetes, and congestive heart failure. We also included other NSAIDs (ibuprofen, naproxen, and aspirin) and non-NSAID analgesics (paracetamol, opioids) in the multivariate model. We stratified analyses by gender and presence of supporting READ codes (surgery, chemotherapy, radiotherapy, or referrals to a specialized oncology clinic). In further subanalyses, we restricted cases to glioblastoma patients, used the combination of all NSAIDs as exposure variable, excluded diclofenac from the group of selective COX-2 inhibitors due to its lower affinity to COX-2, and investigated mutually exclusive use of selective COX-2 inhibitors (i.e., excluding patients using other NSAIDs from the study population).

## Results

We identified 2,469 cases and 24,690 controls in the CPRD database. The mean age ± standard deviation (SD) at the time of glioma diagnosis was 55.3 ± 18.8 years. 44.7% of cases and controls were women. The mean number of years of active history in the database prior to the index date was 11.3 ± 5.2 years for cases and controls. Among patients with known WHO grade of glioma (58.1%), 1,079 patients (75.2%) were diagnosed as glioblastoma.

Table 1 displays characteristics of glioma cases and controls. As with prior data using the CPRD [49], underweight versus normal weight (OR = 0.42; 95% CI = 0.24–0.74), a history of congestive heart failure (OR = 0.58; 95% CI = 0.38–0.87) and a history of diabetes mellitus (OR = 0.81; 95% CI = 0.67–0.97) were related to reduced glioma risk. Current versus never smoking (OR = 0.87; 95% CI = 0.77–0.99) also showed an inverse relation to glioma. By comparison, no associations with glioma were found for rheumatoid arthritis, history of allergies, or use of estrogens in women.

**Table 1:** Characteristics of glioma cases and controls.

Variable	Number of cases (%) (*n* = 2,469)	Number of controls (%) (*n* = 24,690)	OR (95% CI)*
Age (years)*			
0–9	72 (2.9)	728 (3.0)	-
10–19	95 (3.9)	941 (3.8)	-
20–29	113 (4.6)	1,122 (4.5)	-
30–39	182 (7.4)	1,827 (7.4)	-
40–49	273 (11.1)	2,765 (11.2)	-
50–59	536 (21.7)	5,308 (21.5)	-
60–69	608 (24.6)	6,151 (24.9)	-
70–79	450 (18.2)	4,514 (18.3)	-
80–90	140 (5.7)	1,334 (5.4)	-
Sex*			
Men	1,365 (55.3)	13,650 (55.3)	-
Women	1,104 (44.7)	11,040 (44.7)	-
**BMI (kg/m**^**2**^**)**			
< 18.5	13 (0.5)	289 (1.2)	***0*.*42 (0*.*24–0*.*74)***
18.5–24.9	753 (30.5)	7,119 (28.8)	1.00 (reference)
25–29.9	749 (30.3)	7,295 (29.6)	0.97 (0.87–1.08)
≥30.0	401 (16.2)	4,046 (16.4)	0.94 (0.83–1.07)
Unknown	553 (22.4)	5,941 (24.1)	***0*.*84 (0*.*73–0*.*96)***
**Smoking status**			
Never smoker	1,185 (48.0)	11,071 (44.8)	1.00 (reference)
Current smoker	398 (16.1)	4,220 (17.1)	***0*.*87 (0*.*77–0*.*99)***
Past smoker	578 (23.4)	5,784 (23.4)	0.94 (0.84–1.05)
Unknown	308 (12.5)	3,615 (14.6)	***0*.*69 (0*.*58–0*.*82)***
**Comorbidities**			
CHF	25 (1.0)	424 (1.7)	***0*.*58 (0*.*38–0*.*87)***
Diabetes	138 (5.6)	1,674 (6.8)	***0*.*81 (0*.*67–0*.*97)***
Rheumatoid arthritis	24 (1.0)	277 (1.1)	0.86 (0.57–1.32)
Allergies	89 (3.6)	944 (3.8)	0.94 (0.75–1.18)
**Medication use**			
Estrogens^#^			
No prior use	837 (75.8)	8,340 (75.5)	1.00 (reference)
1–14 Rx	177 (16.0)	1,812 (16.4)	1.03 (0.86–1.25)
≥ 15 Rx	90 (8.2)	888 (8.0)	1.04 (0.79–1.36)

*Matching variables: age, sex, general practice, and number of years of active history in the database.

^#^Women only. BMI: Body mass index; CHF: congestive heart failure; OR: Odds ratio, Rx: prescriptions.

Table 2 provides ORs of glioma in association with use of selective COX-2 inhibitors or use of other NSAIDs or non-NSAID analgesics. There was no association between use of selective COX-2 inhibitors and risk of glioma. As compared with no prior use, the OR for 1–9 prescriptions was 1.02 (95% CI = 0.92–1.13), for 10–29 prescriptions it was 1.01 (95% CI = 0.80–1.28), and for ≥30 prescriptions it was 1.16 (95% CI = 0.86–1.55, p-value for trend = 0.408). Results were similar for aspirin and ibuprofen (Table 2).

**Table 2:** Risk of glioma in patients using NSAIDs or other analgesics.

Variable	Number of cases (%) (*n* = 2,469)	Number of controls (%) (*n* = 24,690)	Adjusted OR (95% CI)*^/^^#^
**Selective COX-2 inhibitors**			
No prior use	1,609 (65.2)	16,242 (65.8)	1.00 (reference)
1–9 Rx	710 (28.8)	6,982 (28.3)	1.02 (0.92–1.13)
10–29 Rx	91 (3.7)	939 (3.8)	1.01 (0.80–1.28)
≥ 30 Rx	59 (2.4)	527 (2.1)	1.16 (0.86–1.55)
p-value for trend			0.408
**Aspirin**			
No prior use	2,405 (97.4)	23,954 (97.0)	1.00 (reference)
1–9 Rx	40 (1.6)	481 (2.0)	0.83 (0.60–1.15)
10–29 Rx	11 (0.5)	140 (0.6)	0.80 (0.43–1.50)
≥ 30 Rx	13 (0.5)	115 (0.5)	1.19 (0.66–2.13)
p-value for trend			0.748
**Ibuprofen**			
No prior use	1,676 (67,9)	16,607 (67,3)	1.00 (reference)
1–9 Rx	718 (29.1)	7,353 (29.8)	0.95 (0.86–1.05)
10–29 Rx	53 (2.2)	505 (2.1)	1.03 (0.77–1.39)
≥ 30 Rx	22 (0.9)	225 (0.9)	0.94 (0.60–1.48)
p-value for trend			0.932
**Naproxen**			
No prior use	2,166 (87.7)	21,454 (86.9)	1.00 (reference)
1–9 Rx	277 (11.2)	2,931 (11.9)	0.91 (0.79–1.05)
10–29 Rx	11 (0.5)	203 (0.8)	***0*.*52 (0*.*28–0*.*96)***
≥ 30 Rx	15 (0.6)	102 (0.4)	1.45 (0.83–2.52)
p-value for trend			0.692
**Non-NSAID analgesics**			
**Paracetamol**			*** ***
No prior use	1,291 (52.3)	12,839 (52.0)	1.00 (reference)
1–9 Rx	841 (34.1)	8,417 (34.1)	***0*.*80 (0*.*68–0*.*94)***
10–29 Rx	168 (6.8)	1,674 (6.8)	0.87 (0.66–1.14)
≥ 30 Rx	169 (6.8)	1,760 (7.1)	0.76 (0.55–1.06)
p-value for trend			0.403
**Opioids**			
No prior use	1,319 (53.4)	13,556 (54.9)	1.00 (reference)
1–9 Rx	853 (34.6)	8,106 (32.8)	***1*.*32 (1*.*12–1*.*56)***
10–29 Rx	135 (5.5)	1,405 (5,7)	1.19 (0.88–1.60)
≥ 30 Rx	162 (6.6)	1,623 (6.6)	1.35 (0.96–1.89)
p-value for trend			0.439

*Matching variables: age, sex, general practice, and number of years of active history in the database. ^#^Adjustment for body mass index, smoking, diabetes, congestive heart failure, and all other medications in this table. COX: Cyclooxygenase; NSAIDs: non-steroidal anti-inflammatory drugs; Rx: prescriptions.

We noted that as compared with no prior use, 10–29 prescriptions of naproxen or 1–9 prescriptions of paracetamol were inversely associated with glioma risk (OR = 0.52; 95% CI = 0.28–0.96, OR = 0.80; 95% CI = 0.68–0.94, respectively), but there was no significant trend with increasing numbers of prescriptions (naproxen: p-value for trend = 0.692; paracetamol: p-value for trend = 0.403). Use of 1–9 prescriptions of opiates/opioids was positively associated with glioma risk (OR = 1.32; 95% CI = 1.12–1.56) but again, there was no detectable dose-response relation when investigating increasing duration of drug use (p-value for trend = 0.439) (Table 2). When analysing individual selective COX-2 inhibitors in relation to glioma risk, we found no significant associations with use of etodolac, meloxicam, celecoxib, rofecoxib, etoricoxib, valdecoxib, lumiracoxib, or diclofenac (Table 3). Frequencies of use of the specific COX-2 inhibiting drugs and the grouped variable of “selective COX-2 inhibitors” vary due to successive or combined use of several selective COX-2 inhibitors.

**Table 3:** Risk of glioma in patients using etodoloac, meloxicam, celecoxib, rofecoxib, etoricoxib, valdecoxib, lumiracoxib or diclofenac.

Variable	Number of cases (%) (*n* = 2,469)	Number of controls (%) (*n* = 24,690)	Adjusted OR (95% CI)*^/^^#^
**Etodolac**			
No prior use	2,441 (98.9)	24,477 (99.1)	1.00 (reference)
1–9 Rx	21 (0.9)	180 (0.7)	0.79 (0.17–3.70)
10–29 Rx	5 (0.2)	20 (0.1)	1.77 (0.30–10.42)
≥ 30 Rx	2 (0.1)	13 (0.1)	0.64 (0.15–2.81)
p-value for trend			0.260
**Meloxicam**			
No prior use	2,399 (97.2)	24,006 (97.2)	1.00 (reference)
1–9 Rx	55 (2.2)	565 (2.3)	0.98 (0.73–1.31)
10–29 Rx	11 (0.5)	77 (0.3)	1.37 (0.71–2.66)
≥ 30 Rx	4 (0.2)	42 (0.2)	0.98 (0.34–2.82)
p-value for trend			0.662
**Celecoxib**			
No prior use	2,395 (97.0)	23,983 (97.1)	1.00 (reference)
1–9 Rx	60 (2.4)	613 (2.5)	1.00 (0.75–1.33)
10–29 Rx	10 (0.4)	71 (0.3)	1.51 (0.76–3.00)
≥ 30 Rx	4 (0.2)	23 (0.1)	1.67 (0.57–4.89)
p-value for trend			0.196
**Rofecoxib**	** **	** **	
No prior use	2,407 (97.5)	24,051 (97.4)	1.00 (reference)
1–9 Rx	52 (2.1)	530 (2.2)	0.99 (0.73–1.33)
10–29 Rx	9 (0.4)	88 (0.4)	0.92 (0.45–1.88)
≥ 30 Rx	1 (0.0)	21 (0.1)	0.61 (0.10–3.63)
p-value for trend			0.473
**Etoricoxib**			
No prior use	2,441 (98.9)	24,424 (98.9)	1.00 (reference)
1–9 Rx	26 (1.1)	223 (0.9)	1.12 (0.73–1.71)
≥ 10 Rx	2 (0.1)	43 (0.2)	0.50 (0.12–2.05)
p-value for trend			0.263
**Valdecoxib**	** **	** **	** **
No prior use	2,467 (99.9)	24,663 (99.9)	1.00 (reference)
≥ 1 Rx	2 (0.1)	27 (0.1)	0.80 (0.19–3.36)
**Lumiracoxib**			
No prior use	2,468 (100.0)	24,684 (100.0)	1.00 (reference)
≥ 1 Rx	1 (0.0)	6 (0.0)	1.28 (0.10–16.42)
**Diclofenac**			
No prior use	1,683 (68,2)	16,902 (68,5)	1.00 (reference)
1–9 Rx	676 (27.4)	6,733 (27.3)	1.00 (0.90–1.11)
10–29 Rx	70 (2.8)	690 (2.8)	1.05 (0.81–1.36)
≥ 30 Rx	40 (1,6)	365 (1.5)	1.09 (0.77–1.54)
p-value for trend			0.539

*Matching variables: age, sex, general practice, and number of years of active history in the database. ^#^Adjustment for body mass index, smoking, diabetes, congestive heart failure, all other medications in this table and Table 2 except the combined variable of selective COX-2 inhibitors.

There was no effect modification by sex or when restricting cases (and corresponding controls) to glioblastoma patients or to glioma patients with READ codes for surgery, chemotherapy, radiotherapy, or referrals to a specialized oncology clinic. The results remained similar to those of our main analysis when we conducted analyses of patients with <5 prescriptions of selective COX-2 inhibitors as the non-exposed, when combining all NSAIDs, when excluding diclofenac from the group of selective COX2 inhibitors, or when investigating mutually exclusive use of selective COX-2 inhibitors (data not shown).

## Discussion

To the best of our knowledge, the current analysis is the first observational study to report on use of selective COX-2 inhibitors specifically in relation to the risk of glioma. In contrast to a large number of laboratory studies showing potential preventive effects of selective COX-2 inhibitors on glioma pathogenesis, we found no association between selective COX-2 inhibitor use and glioma.

The relation of NSAID use to risk of glioma or brain cancer has been explored in a number of previous observational studies. Three case–control studies observed a statistically significant inverse association between use of NSAIDs and risk of glioma [40, 41] or glioblastoma [39] Three other case-control studies [34, 38, 42], one prospective cohort study [37], one randomized clinical trial [36], and one recent meta-analysis [35] found no association between NSAID use and brain tumour risk, and two previous cohort studies [43, 44] found an increased risk of brain tumours among NSAID users. Such heterogeneity of results from previous studies may be due to differences in study designs, exposure assessments (i.e., self-reported versus medical record data), underlying drug dosages, durations of use, and adjustments for covariates [35].

None of the aforementioned studies [34–44] investigated the association between use of selective COX-2 inhibitors specifically and glioma risk. A large number of laboratory investigations reported on inhibitory effects of selective COX-2 inhibitors on various stages of glioma pathogenesis [3–6, 8–13, 15, 18–23], prompting clinical trials to investigate selective COX-2 inhibitors as adjuvant therapy for glioma [24–32]. The discrepancy between the laboratory findings and our results may be explained by a number of factors. First, despite mechanistic data regarding protective effects of selective COX-2 inhibitors on glioma development [33], most preclinical studies were designed to investigate therapeutic rather than preventive drug effects. In addition, drug doses used in *in vitro* experiments often exceeded the usual drug concentrations reached in the brain, particularly in experiments involving intratumoral application of selective COX-2 inhibitors or coupling of selective COX-2 inhibitors to nanoparticles aimed at increasing drug concentrations in the tumour [52]. Phase I/II clinical trials were also unable to show a clear benefit of adjuvant therapy with selective COX-2 inhibitors [24, 25, 28–31], even though they were modelled on biological tumour models and used combined therapeutic approaches.

Our null result for selective COX-2 inhibitors and glioma are in line with previous null results for glioma and use of diclofenac, which has a 4-fold higher selectivity for COX-2 than COX-1 [51].

We observed an increased glioma risk in association with short-term use of opiates/opioids. Possible underlying biologic mechanisms remain speculative but may involve opiate-mediated activation of Mitogen-Activated-Kinases [53] or Akt kinases [54]. However, confounding by indication due to opiate or selective COX-2 inhibitors use in patients with pre-existing but undiagnosed glioma or glioblastoma is another possible explanation, despite the fact that we shifted the index date backward in time by one year for cases and controls.

Certain shortcomings of our study warrant discussion. While patients using selective COX-2 inhibitors require drug prescriptions, other NSAIDs such as aspirin, ibuprofen, and naproxen are available over the counter (OTC) in the UK [55]. We therefore may have underestimated NSAID use in patients taking OTC medications, which could have biased the risk estimates towards the null value. However, a prior study showed that prescription-based data adequately reflect use of OTC medications [56]. Also, we had rather small sample sizes in some exposure categories, such as long-term intake of selective COX-2 inhibitors among cases. We were unable to closely control for socioeconomic status, and we were not able to take physical activity or other lifestyle factors possibly related to glioma into account in our analyses, because these variables are not regularly recorded in the CPRD. However, prior studies have not revealed meaningful associations between adulthood lifestyle factors and glioma risk [57, 58]. Our findings may not apply to non-Caucasian populations, because 86% of individuals in our database are Caucasian [59].

Our study has a number of important strengths. The CPRD is a well-established, large-, and validated database [46]. Because we generated cases and controls from a pre-existing database, selection bias is unlikely. Further, recall bias is absent because the data regarding medications and concomitant diseases were collected prospectively. Finally, only patients with an active history of at least three years in the CPRD database were included in the current study in order to increase the likelihood of capturing only newly diagnosed cases and to ensure a sufficiently long history of exposure to selective COX-2 inhibitor use.

In summary, we found no association between use of selective COX-2 inhibitors and the risk of glioma. Further observational studies are warranted to confirm our findings and to help explain the apparent discrepancies between laboratory and observational data.
